# DIRECT CARE STAFF PERSPECTIVES ON THE IMPACT OF PAL CARDS IN CARE DELIVERY

**DOI:** 10.1093/geroni/igac059.440

**Published:** 2022-12-20

**Authors:** Katherine Abbott, Alexandra Heppner, Megan Kelley, Kamryn Kasler, Miranda Kunkel, Kimberly Van Haitsma

**Affiliations:** Miami University, Oxford, Ohio, United States; Miami University, Oxford, Ohio, United States; Miami University, Oxford, Ohio, United States; Miami University, Oxford, Ohio, United States; Miami University, Oxford, Ohio, United States; Pennsylvania State University, University Park, Pennsylvania, United States

## Abstract

This was the third time replicating the Preferences for Activity and Leisure (PAL) Card quality improvement project, but the first conducted entirely during the height of the pandemic. Nursing home providers attempted n=174 PAL Cards and completed n=166 (96%). Feedback from surveys with n=68 staff who came in daily contact with residents (e.g., 26% CNAs, 10% housekeepers, 6% maintenance, 9% dining) were collected by project champions. Staff had worked on average 6 years, indicated that they remember being been told about PAL Cards (81%), noticed them (94%), reported that the information on the PAL Card helped them start a conversation with a resident (79%), and helped them provide care (59%). PAL Cards are a feasible method to communicate important resident preferences to staff who come in daily contact with residents and may not have access to electronic health records. Recommendations for practice will be discussed including staff recommendations for improvements.

